# Assessing Community Health Information Systems: Evidence from Child Health Records in Food Insecure Areas of the Ethiopian Highlands

**DOI:** 10.1007/s10995-020-02943-1

**Published:** 2020-04-28

**Authors:** Kalle Hirvonen, Guush Berhane, Thomas Woldu Assefa

**Affiliations:** 1 International Food Policy Research Institute, C/O ILRI, Bole Sub-City, Kebele no 13, Box 5689, Addis Ababa, Ethiopia; 2 University of Georgia, Athens, USA

**Keywords:** Community health information systems, Ethiopia

## Abstract

**Objectives:**

This study assessed the completeness of child health records maintained and collected within community health information system in Ethiopia.

**Methods:**

A household listing was carried out in 221 enumeration areas in food insecure areas of Ethiopia to determine the presence of a child less than 24-months. This list of children was then compared against the information stored at the local health posts. A household survey was administered to a sample of 2155 households that had a child less than 24-months of age to assess determinants and consequences of exclusion from the health post registers.

**Results:**

Out of the 10,318 children identified during the listing, 36% were found from the health post records. Further analysis based on the household survey data indicated that health posts that had adopted nationally recommended recordkeeping practices had more complete records (p < 0.01) and that children residing farther from health posts were less likely to be found from the registers (p < 0.05). Mothers whose child was found from the registers were more likely to know a health extension worker (p < 0.01), had a contact with one (p < 0.01), and their child was more likely to have received growth monitoring (p < 0.05).

**Conclusions for Practice:**

The incompleteness of the data collected at the health posts poses a challenge for effective implementation of the national health extension program and various complementary programs in Ethiopia.

**Electronic supplementary material:**

The online version of this article (10.1007/s10995-020-02943-1) contains supplementary material, which is available to authorized users.

## Significance

Community Health Information Systems (CHIS) play a key role in the implementation of various health and nutrition programs that target young children and pregnant women. Despite this, the quality and completeness of these data are largely unknown. This study assesses the quality of child health data collected within CHIS in Ethiopia, the second most populous country in Africa. The finding that only 36% of the children less than 24 months of age were found from the health post records suggest that many rural children are not fully exposed to the health and nutrition services provided by the community health workers.

## Introduction

Health Information Systems (HIS) play a key role in the design and implementation of health care policies (WHO 2007). A strong and well-functioning HIS is paramount for identifying and addressing public health emergencies as well as inequalities in access to health and nutrition services (AbouZahr and Boerma 2005; Nolen et al. 2005; Thieren 2005). Reliable HIS is also important in order to monitor progress towards achieving the sustainable development goals set by the United Nations in 2015 (United Nations 2015), many of which focus on infants and young children as well as pregnant and lactating women.

HIS has multiple functions (WHO 2008). The individual or patient level data are used by health care professionals for clinical decision making. Data collected at the health facility level are used to make decisions related to staffing and other resource needs. The individual and facility level data can then be aggregated to population level to guide decision making on public health issues, for example related to equity in the health care system. If these aggregated data are generated in a timely fashion, they can also be used for public health surveillance to identify disease epidemics.

In low-income countries, most people reside in rural areas and therefore a large part of the routine health information is collected at the community level, typically by community health workers. Despite the central role of such Community Health Information Systems (CHIS) in the implementation of national and sub-national health care programs, there exists limited evidence on the overall quality of these health records. The available evidence from sub-Saharan Africa indicates that the data collected through CHIS is of variable quality. A recent study from one district in Rwanda compared household survey data with routine health data collected by the community health workers (Mitsunaga et al. 2015). This study concluded that the data collected by community health workers was largely of good quality. In Malawi, the data quality collected by the health surveillance assistants was markedly improved after adjusting the data collection instruments and reducing the workload of the assistants (Admon et al. 2013). A recent study focusing on vital statistics in the Oromia region of Ethiopia found that only 30% of births and about 21% of under-five deaths were reported by community health workers (Amouzou et al. 2015).

In Ethiopia, the focus of this study, CHIS is maintained by the Health Extension Workers (HEWs) who store and compile the data at the health posts, typically in a non-electronic format. While the Federal Ministry of Health and independent consultants have recently raised concerns about the quality of the data collected under CHIS (Bossuyt 2017; FMOH 2015), there has not been systematic assessments of CHIS. Using geographically widespread survey data, we take a first step toward this direction by assessing the completeness of recordkeeping of child health data within CHIS. We focus on households with infants and young children that are in need of critical family health services provided by the HEWs, including medical care, monitoring and counselling. Using survey data collected by the authors, this article addresses two research questions. First, how many of the children residing in our surveyed areas are found from the health post registers? Second, what implications does the exclusion from these records have in terms of exposure to the services provided by the HEWs?

## Material and Methods

### Study Setting

Ethiopia’s Health Extension Program (HEP) has been implemented by the Government of Ethiopia since 2002 and now covers nearly all *woredas* (districts) of the country. As an integrated program, the HEP focuses on four primary areas: hygiene and environmental sanitation, disease prevention and control, family health services and health education and communication (Workie and Ramana 2013). The HEWs stationed at the *kebele* (sub-district, lowest administrative unit) health posts play a key role in implementing the HEP activities. The health posts are built by the government and typically located at the kebele center. The infrastructure at the health posts is often rudimentary with many lacking access to reliable electricity, phone services and clean water (Bilal et al. 2011). There are typically two HEWs working in one health post and together these government employees are expected to reach approximately 5000 individuals (Lemma and Matji 2013). Each health post is supervised by a health center. Five health posts and one health center form a primary health care unit that serves ~ 25,000 people (Bilal et al. 2011). HEWs receive assistance from Health Development Army (HDA); a group of volunteers residing in the villages and supporting the HEWs.

**Fig. 1:**
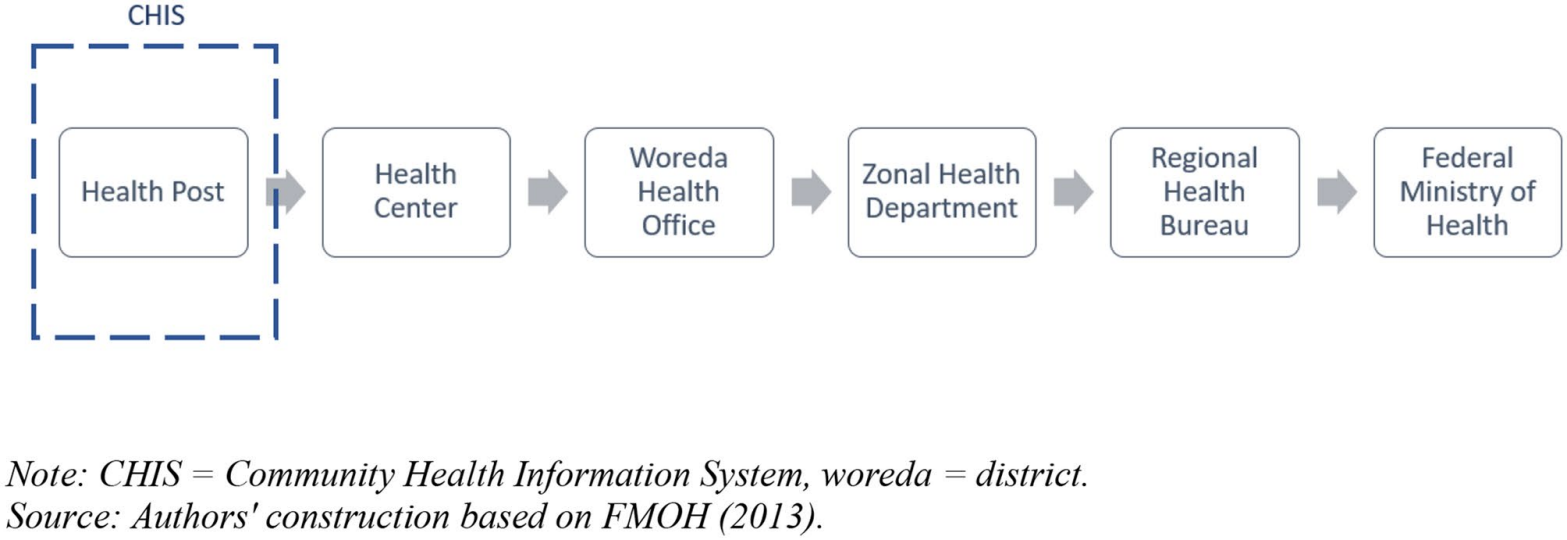
Information flow in Ethiopia's Health Management Information System. *CHIS* community health information system, *woreda* district. Source: Authors' construction based on FMOH (2013)

The Health Management Information System (HMIS) designed by the Ministry of Health plays a key role in facilitating data analysis to aid strategic planning. Since most Ethiopians reside in rural areas, the CHIS is a key component in the HMIS. Figure 1 shows the information flow within Ethiopia's Health Management Information System. HEWs collect data from their clients and store them at health posts. This is largely a paper based system, though efforts are under way to move to an electronic record keeping system in the near future (Advancing Partners and Communities 2019). Data from health posts are then sent in paper format to the health center once a month. The staff at the health centers compile the data and submit them to the woreda health office. At the woreda health office, the data form different health posts and centers are compiled and entered into a digital format. After this, these digitized data are sent to the zonal health department, which passes them on to the regional health bureau. The Federal Ministry of Health then gets the data from regional health bureaus.

At the health posts, the HEWs use three main record keeping instruments to collect data within the CHIS: Master Family Index, Family Folders and Individual Health Cards. The Master Family Index is a booklet listing all households residing in the kebele. Each household residing in the catchment area (kebele) should have a Family Folder at the health post (FMOH 2011). The Family Folder contains information on household demographics and other household characteristics relevant to health and nutrition; information on household's Water, Sanitation and Hygiene (WASH) infrastructure and the use of insecticide treated nets to prevent malaria. Inside the folder, there should be a Health Card for each household member who is five years or older. The information on the younger children are kept in the mother's Health Card until they turn five. This health card contains information on the provision of family planning services, immunization and growth monitoring, among others. As such, CHIS plays a central role in the HEWs work and implementation of the HEP at the community level.

### Data

This assessment was carried out as a part of a larger evaluation of the Ethiopia's flagship safety net program—the Productive Safety Net Program (PSNP). The program currently operates in more than 300 woredas and supports about eight million people. Most PSNP beneficiaries receive cash or food payments against public works that take place over a six-month period, typically between January and June. Eligible households with limited labor capacity receive unconditional payments. The core objective of the PSNP has been to improve food security and prevent asset depletion in chronically food insecure areas of the country. Recently, the program added an increased emphasis on improving nutritional status of young children and their mothers. Therefore, the purpose of the evaluation was to assess the impact of PSNP on child and maternal nutrition. To this end, we conducted two baseline (or pre-intervention) and two endline (or post-intervention) surveys in PSNP woredas in the Ethiopian highland regions: Amhara, Oromia, Southern Nations, Nationalities, and People's Region (SNNP) and Tigray. The survey focused on households with a child less than 24-months of age. The first baseline survey was administered between the end of February and the beginning of April in 2017 and the second baseline in August 2017. The two endline surveys were administered during the same months in 2019.

The current study uses the data from the first baseline survey in February-April 2017. Table 1 provides an overview of the sampling and data collection procedures. The sampling for this survey was done in stages. First, 88 woredas were randomly selected from the full list of PSNP woredas in Amhara, Oromia, SNNP and Tigray regions. Three kebeles were randomly selected from each woreda. For census work purposes, the Central Statistical Agency (CSA) of Ethiopia divides all kebeles in the country into enumeration areas (EA) that are roughly of equal size, typically containing about 200 households. We used the CSA's EA maps to randomly select one EA from each kebele and then conducted a full listing of households residing in the EA. The primary purpose of this household listing was to form a sampling frame; to identify all households in the EA that had a child less than 24 months of age.

**Table 1:** Diagram of the sampling and data collection procedures

Action	Total
Randomly select 88 woredas	88 woredas
After woreda selection	
Randomly select 3 kebeles from each woreda	264 kebeles
After kebele selection	
Randomly select 1 EA from each kebele	264 EAs
After the EA selection	
Conduct a household listing survey in EA	46,866 households, out of which 10,318 had a child < 24 months
After household listing survey	
Visit the health post in each kebele and try to find the children identified in the household listing survey from the health post records	221 health posts
Randomly select 10 eligible households in each EA in-depth interview and conduct in-depth interviews with the selected households	2640 households

*EA* enumeration area

The listing and other data collection activities in each EA were carried out by a survey team consisting of one supervisor and four enumerators (22 supervisors and 88 enumerators were deployed in total). The enumerators carefully divided the EA into four areas and visited each household in the EA. A listing of one EA was typically done in one day. If nobody from the household was present at the time of the visit, the enumerators collected the required information from their neighbors or knowledgeable village guides assigned for this purpose. A total of 46,866 households residing in the 264 EAs were listed. Out of these, 10,318 households reported that they had at least one child less than 24 months of age. The presence of more than one child with less than 24 months in the same household was rare. In such cases, the enumerators were instructed to note the information of the youngest child of the household head and the spouse. Polygamy is not practiced in the localities that were surveyed.

After the EA listing was completed, the survey team was left with two tasks. One enumerator took the list of names of the children identified in the EA listing to the kebele health post. At the health post, this enumerator and a HEW together compared this EA list to the information in the health information records available at the health post: Master Family Index, Family Folders and the individual Health Cards as well as any other material available at the health post.

The remaining enumerators began the household level survey. This in-depth household survey was administered to 10 eligible households that were randomly selected from each EA using the information collected during the listing. A household was eligible if it had a child less than 24-months of age. We further stratified the sample so that roughly half of the selected households were PSNP beneficiaries and half of them were poor (based on their own assessment) but not benefitting from the PSNP. The survey collected information on basic household characteristics (household demographics, education attainment, wealth levels, etc.), exposure to health and nutrition services, health status, anthropometry, and so on.

A total of 2635 households with a child less than 24 months of age were interviewed in 88 woredas and 264 kebeles in Amhara, Oromia, SNNP and Tigray. Of note is that the in-depth interview provided an opportunity to validate the information collected during household listing. Only in extremely rare cases (less than one percent), the household selected for the survey did not have a child in the specified age range (0–23 months). In such cases, a replacement household was drawn from the EA household list.

This current study focused on a sub-sample of 221 kebeles in which the health post was also visited by the survey team. The enumerators were unable to visit all 264 health posts because some kebeles did not have a health post or because the health extension workers were absent due to training, leave or other reason. The number of households interviewed in these 221 kebeles was 2218. After removing households with missing observations, the final household sample used in the regression analysis is 2155 households. Given the focus on poor households, this household sample is not representative of the EAs or kebeles in which the sample was drawn.

### Ethical Considerations

All data were collected via face-to-face interviews using structured questionnaires and verbal informed consent was obtained from all participants. Ethical approval was obtained from the Institutional Review Board of the International Food Policy Research Institute.

## Methods

All statistical analyses were conducted using Stata, version 15.0 (StataCorp, Texas). The analysis took place in stages. In the first stage, we used the listing data of 10,318 households with a child less than 24 months and reported the share of the children found in the health post registers.

In the second stage, we restricted the analysis to households which were randomly selected for the in-depth household survey. Multivariate probit methods were used to assess the child, household, and HEW level determinants of inclusion into the health post records. These same methods were used to test whether the inclusion in the registers was correlated with exposure to health and nutrition services. This was proxied based on mothers' self-reports whether they knew a HEW working in her kebele and whether or not the mother had had a contact with HEW in the past 3 months. Moreover, the HEWs are tasked by the government to monitor the physical growth of children less than 24 months of age in their kebele (GFDRE 2013, 2016). This growth monitoring involves measuring the height, weight and mid-upper arm circumference (MUAC) of the children. The association between exposure to growth monitoring and inclusion in the health post records was also assessed.

### Variables

We created a binary variable that obtained one if the child was found in the health post registers and zero otherwise. This variable was used as the dependent variable in the regression models that explored the determinants of inclusion in the registers and as the key independent variable in the models that tested whether the inclusion was associated with the likelihood of receiving services provided by the HEWs.

The independent variables used in the first regression model were sourced from the interviews at the health posts and household interviews. During the health post visit, the enumerators took note whether the Master Family Index or the Family Folder existed at the health post. We created binary variables capturing one if they did and zero otherwise. Using the health post level data, we also constructed a variable capturing the number of years the HEW had worked at the health post. Moreover, using the latitude–longitude coordinates of the health post and the households, we calculated household's distance to the health post. This variable was measured in kilometers. Child and household level variables were constructed from the household level data. Child's age was measured in months and sex using a binary variable that obtained one if the child was male and zero if female. Place of birth was captured using a binary variable obtaining value one if the child was born home and zero if elsewhere. Mother's age and level of education were both measured in years. The regression model also included variables capturing household size, a binary variable obtaining value one if the household had other children who are less than 5 years of age (and zero if not). Household wealth was proxied with the number of tropical livestock units (TLU) owned by the household and with a binary variable whether the household benefitted from the PSNP program. A set of binary variables for each four regions were constructed and included in the model.

The second set of regressions used three different binary dependent variables to measure household's exposure to health and nutrition services. First, we constructed a binary variable obtaining one if the mother of the child reported to know a health extension worker in their kebele (and zero otherwise). Second, another binary variable was constructed to capture if mothers had a contact with a HEW in the past 3 months. The third binary variable obtained a value one if the mother reported that the child had received growth monitoring in the past 3 months (and zero otherwise). The main independent variable of interest here was the binary variable capturing child's inclusion in the health post records. The other independent (control) variables were the same as used in the first regression.

## Results

**Fig. 2:**
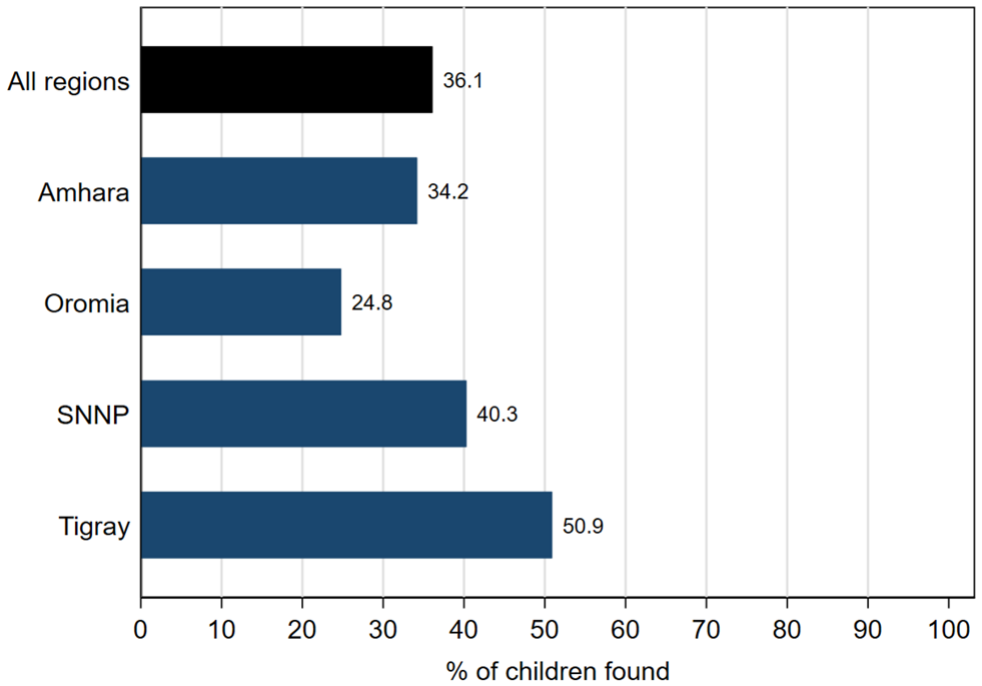
Percent of children under 24 months found in the health post records, by region

Based on the EA listing data, only 36 percent of the children in the PSNP highland kebeles were found in the health post records (Fig. 2). Regional disaggregation revealed that children in Tigray (51%) and SNNP (40%) regions were more likely to be found in the health post records, compared to children in Amhara (34%) and Oromia (25%) regions.

Table 2 provides the summary statistics—means, standard deviations, minimums and maximums—for the variables used in the analysis based on household survey data. The percent of children found from the records was slightly higher in this sample compared to the data based on the EA listing (39.0% vs 36.1%). The MFI system existed in 82.2% and the Family Folders were in use in 85.5% of the health posts. Table S1 in the Supplementary further breaks these statistics by region. We see that the adoption rates of these health recording systems were somewhat higher in Tigray and SNNP compared to Amhara and Oromia. Finally, less than half of the children were born in a health facility (at a health post, health center or hospital). Table S2 in the Supplementary material expands this information and disaggregates these statistics by region.

**Table 2:** Summary statistics for the variables used in the regression analyses (N = 2155)

	Mean	Std. dev	Min	Max
= 1 if child is found from the health post records, 0 if not found	0.390	0.488	0	1
Health post or Health Extension worker characteristics				
= 1 if Master Family Index exists at the health post, 0 otherwise	0.822	0.382	0	1
= 1 if family folders exist at the health post, 0 otherwise	0.855	0.352	0	1
Household's distance (in km) to the health post	2.174	1.788	0.02	9.04
Number of years the HEW has worked in the kebele	4.231	3.623	0	12
Child or household characteristics				
Child's age in months	11.36	6.71	0	23
= 1 if index child is male, 0 if female	0.514	0.500	0	1
= 1 if the child was born home, 0 otherwise	0.524	0.500	0	1
Mother's age (in years)	28.83	6.49	16	50
Mother's education (in years)	0.852	2.237	0	13
Household size	5.729	1.936	2	16
= 1 if household has other less than 5-year-old children, 0 otherwise	0.593	0.491	0	1
Number of tropical livestock units (TLU) owned by the household	2.999	3.731	0	65.4
= 1 if the household benefitted from PSNP in 2016	0.386	0.487	0	1
Binary regional variables				
= 1 if child is in Tigray region, 0 otherwise (reference category)	0.213	0.410	0	1
= 1 if child is in Amhara region, 0 otherwise	0.262	0.440	0	1
= 1 if child is in Oromia region, 0 otherwise	0.244	0.430	0	1
= 1 if child is in SNNP region, 0 otherwise	0.280	0.449	0	1
Variables capturing exposure to health and nutrition services				
= 1 if mother knew a health extension worker, 0 otherwise	0.794	0.404	0	1
= 1 if mother had contact with HEW in past 3 months, 0 otherwise	0.373	0.484	0	1
= 1 if child's growth was monitored in the past 3 months, 0 otherwise	0.282	0.450	0	1

Table 3 provides the probit model estimates that assess the determinants of inclusion in the health post records. The adoption of the family folder system was associated with more complete records: children residing in kebeles where the health post uses the family folder system were, on average, 21 percentage points more likely to be found in health post records. Considering that 39 percent of the sampled children were found from the health post records, this corresponds to nearly 54% increase in the likelihood that the child is found in the registers. The existence of the Master Family Index at the health post was not associated with increased likelihood that the child is found from the records. Household's distance to the health post was strongly correlated with the likelihood that the child is found in the health post registers: each kilometer decreases the likelihood of the child is found in the health post records by 3.2 percentage points, on average. Therefore, a child residing 5 km away from the health post is, on average, 16 percentage points—or 41 percent—less likely to be found in health post records. HEWs work experience in the kebele was not found to be associated with the likelihood that the child was found in health post records.

**Table 3:** Determinants of the probability that child is found from the health post records (marginal effects from probit regressions)

	(1)
*Number of observations (1 child per household):*	*2,155*
*Dependent variable:*	*Child is found from the health post records*
Master Family Index exists at the health post	0.032
	(0.074)
Family Folders exist at the health post	0.208***
	(0.076)
Household's distance (in km) to the health post	− 0.032***
	(0.012)
Number of years the HEW has worked in the kebele	0.002
	(0.006)
Child's age in months	− 0.004**
	(0.002)
Child is male	0.019
	(0.020)
Child was born home	− 0.040
	(0.030)
Mother's age (in years)	0.001
	(0.002)
Mother's education (in years)	0.003
	(0.005)
Household size	− 0.010
	(0.007)
Household has other children who are less than 5 years of age	0.051*
	(0.026)
Number of tropical livestock units (TLU) owned	0.004
	(0.004)
Household benefitted from PSNP in 2016	0.018
	(0.026)
Amhara region	− 0.089
	(0.056)
Oromia region	− 0.195***
	(0.065)
SNNP region	− 0.106*
	(0.059)
Tigray region	(reference)

The non-italicized rows indicate variables, coefficients and standard errors. The italicized rows provide additional information about the model; dependent variable and the sample size

Reported coefficients are marginal effects from a probit model. Standard errors are reported in parentheses, and are clustered at the health post (kebele) level. The standard errors for the marginal effects are calculated using the Delta Method

*, ** and *** indicate significance at the 10%, 5% and 1% level, respectively

Regarding child and household characteristics, older children were somewhat less likely to be found in the records: a 12-month increase in child's age was associated with a 5-percentage point decrease in the likelihood that the child is found in the records. Child's sex or birth place were not found to be statistically significant determinants. Similarly, parental characteristics (age or level of education), household size, wealth (number of tropical livestock units they own) and PSNP beneficiary status were not statistically significant determinants. Children residing in households that had other young children are somewhat more likely to be found in the health post records. However, this coefficient is only statistically significant at the 10% level.

Table 4 shows the probit model estimates on whether incomplete information at the health post registers was associated with services provided by the HEWs. In column 1, we see that mothers whose child was found in the health post registers were 7.7 percentage points more likely to know a HEW, on average. Since 79% of the mothers did know a HEW working in their kebele (Table 2), this translates into a 10% increase in the likelihood that the mother knew a HEW. In column 2, we assess how the inclusion in the records was associated with contact with the HEW. As before, the estimated coefficient is highly statistically significant and positive. Mothers whose child was found in the registers were nearly nine percentage points—or 24%—more likely to have had a contact with a HEW in the past three months, on average. In column 3 we assess how children's exposure to growth monitoring was correlated with inclusion in the health post records. All else equal, we see that children who were found in the registers were, on average, 4.4 percentage points—or 16%—more likely to have received growth monitoring in the past three months compared to other children.

**Table 4:** Association between household's exposure to health and nutrition services and binary indicator of child is found from the health post records

	(1)	(2)	(3)
*Number of observations:*	*2,155*	*2,155*	*2,147*
*Dependent variable:*	*Mother knows health extension worker*	*Mother had had contact with HEW in past 3 months*	*Child's growth was monitored in the past 3 m*
Child is found from the health post records	0.077***	0.089***	0.044**
	(0.019)	(0.023)	(0.022)
Control variables included?	Yes	Yes	Yes

The non-italicized rows indicate variables, coefficients and standard errors. The italicized rows provide additional information about the model; dependent variable and the sample size

Each column reports coefficients from a probit model with different dependent variables but same set of independent variables Coefficients are marginal effects from a probit model. Standard errors are reported in parentheses, and are clustered at the health post (kebele) level. The standard errors for the marginal effects are calculated using the Delta Method

*, ** and *** Indicate significance at the 10%, 5% and 1% level, respectively

Table 5 reports the unadjusted and adjusted service exposure prevalence. The former are simple prevalence estimates calculated from the raw data. The latter were constructed using the predictions from the regressions reported in Table 4 that control for differences in various child, household and HEW characteristics. According to the adjusted means, 87% of the mothers whose child was found in the health post registers knew a HEW working in their kebele. This (adjusted) percentage drops to 79% among mothers whose child was not found in the registers. Similarly, 42% of mothers whose child was registered at the health post did have a contact with a HEW in the past 3 months. The corresponding figure among mothers whose child was not registered was 32%. For growth monitoring, the adjusted percentages are 29 and 25%, respectively.

**Table 5:** Predicted probabilities that the household is exposed to health and nutrition services

	Mother knows health extension worker		Mother had had contact with HEW in past 3 months		Child's growth was monitored in the past 3 months	
	N = 2,155		N = 2,155		N = 2,147	
	Unadjusted	Adjusted	Unadjusted	Adjusted	Unadjusted	Adjusted
Child is in records (%)	87.0	86.9	46.3	41.6	32.3	29.2
Child is not in records (%)	74.6	78.9	31.5	32.1	25.6	24.6
Difference (%p)	12.4***	8.0***	14.8***	9.5***	6.6***	4.6**

*, ** and *** Indicate significance at the 10%, 5% and 1% level, respectively. %p refers to percentage point. Predictions from the adjusted model is based on the probit regression reported in Table 4

## Discussion

Despite considerable progress over the last two decades, child health and nutrition outcomes in Ethiopia are poor; about 38 percent of children under five are stunted (short for their age) and diarrheal and other infection risks remain high (Golan et al. 2019). The community health workers play a key role in implementing the national health extension program and various large-scale nutrition programs in the country. It is therefore worrying that the records kept at the health post in rural Ethiopia are incomplete when it comes to infants and young children. Our study also finds that children who are not found in the health post registers are less likely to be exposed to the services provided by the health extension workers.

This study has limitations. First, while the data used in this study is geographically widespread covering 221 health post in four regions of Ethiopia, these findings should not be interpreted to represent the situation in the entire country. Second, we only assessed the completeness of the information stored at the health posts. Future research should also be directed to understanding the other dimensions of data quality such as accuracy and consistency. Third, this is an observational study and therefore one should not attribute causality to findings regarding the determinants and consequences of exclusion from the health post registers.

With these limitations in mind, the findings reported in this study have important implications to policy. First, effective implementation of health and nutrition programs is likely to be challenging in the absence of complete information about the children residing in the kebeles. Perhaps even more worrying is the finding that the data collected at the health posts and fed into the national health information system are less likely to include children from remote, less well-off families. Moreover, mothers whose children were not found in the health post registers were less likely to know and have contact with HEWs. Second, we also find that health posts that had adopted the recommended record keeping practices had more complete records. While our methods do not allow us to make causal statements, this finding suggests that significant improvements could be made by making sure that Family Folder system are used at the health posts. Shifting to more efficient record keeping systems, for example, to electronic (Fraser et al. 2005) or mobile phone based applications (Medhanyie et al. 2017) could result in further improvements. However, limited access to reliable electricity and poor mobile phone network at many health posts are major barriers for scaling up these approaches. Third, reaching the more remote – and therefore less well-off (Stifel and Minten 2017) —households may need different strategies. Previous studies suggest that extension workers in Ethiopia are already overburdened with multiple activities (Mangham-Jefferies e t al. 2014; Tilahun et al. 2017). If so, more resources may be needed at the health posts. This could mean hiring more HEWs or strengthening the coordination between the HEWs and the HDA so that the HDA members can actively report about births taking place in their communities to their health post.

Finally, this study also has lessons for researchers. Given the incompleteness, and more importantly, non-randomness of the information obtained from the health posts, we strongly caution against using health post records in Ethiopia as a sampling frame. Indeed, results based on such sampling—or on the information available at the health posts (e.g. data on heights and weights of children)—are very likely to be biased. Considering the findings presented in this study, we advise researchers to conduct a full census (listing) of the study area before sample selection.

## Electronic supplementary material

Below is the link to the electronic supplementary material.

Supplementary file1 (DOCX 17 kb)

## Abbreviations

HIS: Health information system
CHIS: Community health information system
CSA: Central statistical agency
EA: Enumeration area
HDA: Health development army
HEP: Health extension program
HEW: Health extension worker
MFI: Master family index
MUAC: Mid-upper arm circumference
PSNP: Productive safety net program
SNNP: Southern nations, nationalities, and peoples' region
TLU: Tropical livestock units
WASH: Water, sanitation and hygiene
