# Supplementary material for: Assessing Community Health Information Systems: Evidence from Child Health Records in Food Insecure Areas of the Ethiopian Highlands
Source: Matern Child Health J. 2020 Apr 28;24(8):1028–37. doi: 10.1007/s10995-020-02943-1 (PMC7320934; doi:10.1007/s10995-020-02943-1)
Supplement: Supplementary file 1 — Supplementary file1 (DOCX 17 kb) [file 10995_2020_2943_MOESM1_ESM.docx]

**Supplementary material**

**Supplemental Table S1: Percent of health posts that had a Master Family Index (MFI) or Family Folders (FF), by region (N = 220)**

|  | **N = 220** | **N = 220** |
| --- | --- | --- |
| **Region** | **MFI exists (%)** | **FF exists (%)** |
| Amhara | 82.5 | 78.9 |
| Oromia | 66.7 | 81.5 |
| SNNP | 88.5 | 95.1 |
| Tigray | 91.7 | 85.4 |
| **All regions** | **82.3** | **85.5** |

*Note: information is missing for one health post.*

**Supplemental Table S2: Birth places, by region (N = 2,155 children; 1 per household)**

| **Region / location** | **Health post** | **Health center** | **Hospital** | **Home** | **Other** |
| --- | --- | --- | --- | --- | --- |
| Amhara | 5.3% | 27.4% | 2.5% | 63.4% | 1.4% |
| Oromia | 9.9% | 12.4% | 5.3% | 72.2% | 0.2% |
| SNNP | 5.6% | 39.2% | 7.0% | 45.9% | 2.3% |
| Tigray | 9.1% | 54.3% | 9.3% | 24.8% | 2.4% |
| **All regions** | **7.3%** | **32.8%** | **5.9%** | **52.4%** | **1.6%** |
